# Protective activity of *Malus doumeri* leaf extract on H_2_O_2_-induced oxidative injury in H9C2 rat cardiomyocytes

**DOI:** 10.3389/fcvm.2022.1005306

**Published:** 2022-09-16

**Authors:** Yi Shen, Zheng Shen, Ping Li, Zhangrong Chen, Bo Wei, Danan Liu, Xiaoyun Si, Jiayi Pan, Daiqin Wu, Wei Li

**Affiliations:** Department of Cardiovascular Medicine, The Affiliated Hospital of Guizhou Medical University, Guiyang, China

**Keywords:** *Malus doumeri* leaf, oxidative toxicity stress, hydrogen peroxide, SOD, HPLC

## Abstract

In this study, *Malus doumeri* leaf extract (MDLE) was used to test its anti-oxidation capacity *in vitro*, it has been preliminarily analyzed for H_2_O_2_-induced oxidative damage in H9C2 cells and its main active components. The antioxidant capacity through DPPH (1, 1-Diphenyl-2-Picrylhydrazyl), ABTS^+^• [2,2,2'-azino-BIS-(3-ethylbenzo-thiazoline-6-sulfonic acid)] radical ion, •OH (hydroxyl radical), and $•{{\text{O}}_{2}}^{-}$ (superoxide anion) were determined *in vitro*. The proliferation of H9C2 cells was examined by MTT [3-(4,5-Dimethyl-2-thiazolyl)-2,5-diphenyl-2-H-Tetrazolium bromide]. MDA (malondialdehyde), SOD (superoxide dismutase), CAT (catalase), GSH (glutathione), and GSH-Px (glutathione peroxidase) were determined by colorimetry. Apoptosis induced by oxidative damage was detected by flow cytometry. The mRNA expression of antioxidant related genes of SOD, CAT, GSH, and GSH-Px were checked by qRT-PCR (quantitative real-time polymerase chain reaction). The MDLE main active components were analyzed by HPLC (high-performance liquid chromatography). MDLE had significant scavenging effects on DPPH, ABTS^+^•, •OH, and superoxide anion radicals in a concentration-dependent manner. H_2_O_2_ treatment could significantly lead to oxidative stress injury of H9C2 cells, and MDLE treatment significantly improved the degree of H9C2 cell damage, and showed a positive correlation with concentration. MDLE can also reduce apoptosis caused by oxidative damage. MDLE treatment could significantly reduce MDA content and increase CAT, SOD, GSH, and GSH-Px contents and expression. In addition, by HPLC analysis, the following six bioactive components were detected from MDLE: chlorogenic acid, isoquercitrin, quercetin, baicalin, and phloretin. Therefore, MDLE has a good protective effect on myocardial cells.

## Introduction

**Figure 1:**
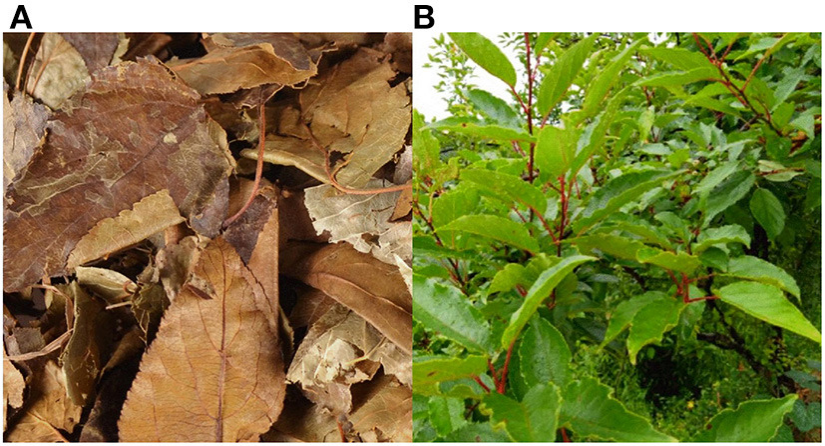
Observation diagram of *Malus doumeri* leaf, **(A)** dry leaves, **(B)** plant leaves.

Plant drinks, including tea, originated from the Bayu region in south China and were used as medicinal drink (1). Tea contains many active compounds such as tea polyphenols, so that tea has strong free radical scavenging and reducing activities (1). *Malus doumeri* leaf belongs to another type of tea, that is, a plant that does not belong to the *genus Camellia* in the Theaceae family. It is produced in the Yangtze River Basin, China, and is mainly refined and processed from the young leaves of local wild *Malus doumeri*. Its leaves contain various beneficial amino acids, flavonoids, and so on (2, 3) (Figure 1). Since the leaves grow in an original ecological environment, the picked leaves can be dried directly under appropriate temperature and humidity, active substances are produced through complex chemical changes triggered by a special fermentation process. Studies have found that *Malus doumeri* leaf has a variety of health care effects, such as blood lipids, relieving nerves, anti-aging, strengthening teeth, inhibiting inflammation, preventing cancer, and improving immunity (4, 5).

Reactive oxygen species (ROS) are the main cause of oxidative stress. Under physiological conditions, the concentration of ROS is very low. It is an important substance involved in signal transduction and can regulate the production of immune inflammatory factors in the body. At the same time, peroxides can induce apoptosis (6, 7). ROS can attack proteins, oxidize amino acids, change their structure, and make them lose their physiological functions. It can oxidize lipids on the surface of cell membranes, changing their structure and function. It can also modify bases and interfere with the function of genetic material (8). In the presence of Fe^2+^, H_2_O_2_ generates hydroxyl radicals (·OH) with strong oxidizing ability, and triggers more other reactive oxygen species to achieve the degradation of organic matter, thereby causing oxidative damage to the body (9). Therefore, this study examined the protective effect of different concentrations of MDLE on oxidative stress-injured cells through H_2_O_2_-induced H9C2 cell injury model. Moreover, the preliminary analysis of potentially active components was conducted to provide some reference for the effect of MDLE on human chronic diseases.

## Materials and methods

### *Malus doumeri* leaf extract preparation

The *Malus doumeri* leaf was produced in Honghua village, Sanxi Township, Wushan County, Chongqing, China, it was produced in 2021, the freeze-dried *Malus doumeri* leaf (Chongqing Wushan Xiajiang Tea Industry Co., Ltd, Chongqing, China) was crushed, ground, and screened, and then a certain amount of *Malus doumeri* leaf powder was precisely weighed into a beaker. Seventy percent ethanol (v/v) was added to the liquid to the material at a 20:1 ratio (w/w), and incubated at 60°C in a water bath for 2 h. This procedure was performed twice. Remove water and ethanol from the resin liquid by rotary evaporator (steam until no liquid flows in the beaker), and dry at 60°C for 48 h; the dried sample was removed, ground, and weighed, then stored in an EP tube and stored in 4°C for later use.

### Hydroxyl radical scavenging experiment

Salicylic acid colorimetric method (10). Take 10 mL corkscrew tube, add 2 mL MDLE aqueous solution of different concentrations (0.2, 0.6, and 1.0 mg/mL) (11), then add 2 mL 9 mmol/L ethyl-salicylic acid solution and 1 mL 9 mmol/L ferrous sulfate solution (prepared with ferrous sulfate seven water, Solarbio Life Science, Beijing, China) successively. Finally, 2 mL 8.8 mmol/L hydrogen peroxide (Solarbio Life Science) was added to initiate the reaction. The samples were incubated at 37°C in a water bath for 30 min, and the OD at 510 nm was measured. Three independent OD measures were performed per group (Themo Genesys10s, ThermoFisher Scientific, Waltham, MA, USA).

### DPPH free radical scavenging assay

0.5 mL MDLE aqueous solution with different concentrations (0.2, 0.6, and 1.0 mg/mL) was added to 2 mL DPPH ethanol solution (0.33 mM, Solarbio Life Science) (11), and incubated in the dark at room temperature for 30 min. The 517 nm value of absorbance in the solution was tested (12), three parallel in each group (Themo Genesys10s, ThermoFisher Scientific).

### ABST clearance experiment

In a 5 mL stopper tube, 1 mL ABTS (2,2,2′-azino-BIS-(3-ethylbenzo-thiazoline-6-sulfonic acid, Solarbio Life Science) free radical working solution and 0.4 mL MDLE solution with different concentrations (0.2, 0.6, and 1.0 mg/mL) were added (11). The tube was filled with solvent, and left in darkness for 30 min, and the OD value at 734 nm was measured. Samples were prepared in triplicate. For the ABTS free radical working solution preparation, 3 mg ABTS was dissolved into 0.8 mL double steamed water to prepare the Liquid A. For Liquid B, 1 mg potassium persulfate was dissolved in 1.5 mL double steamed water. Then, 0.2 mL of liquid A and liquid B were mixed, oxidized in darkness for 12 h, and diluted to A with anhydrous ethanol and determined at OD_734nm_ (Themo Genesys10s, ThermoFisher Scientific) (13).

### Superoxide anion removal experiment

The pyrogallol autoxidation method was used for superoxide anion removal experiment (14). In a 10 mL corkscrew tube, 1 mL MDLE aqueous solution (0.2, 0.6, and 1.0 mg/mL) was mixed with 4.5 mL 0.1 mol/L TRIS-HCl buffer solution (pH = 8.2, Solarbio Life Science), and reacted at 37°C in a water bath for 20 min, then allowed to cool to 25°C. Then 0.4 mL of 50 mmol/L pyrolenol solution (Solarbio Life Science) was placed in a water bath at 37°C for 5 min, and then 0.1 mL of 8.0 mol/L concentrated HCl was added immediately to stop the reaction. All samples were prepared in duplicate.

#### H9C2 cell culture

The H9C2 cells (National Collection of Authenticated Cell Cultures, Shanghai, China) were recovered and inoculated in DMEM medium (high glucose, containing 10% fetal bovine serum and 1% penicillin-streptomycin diabody solution, Solarbio Life Science), and cultured at 37°C in a 5% carbon dioxide environment.

#### Cell viability assay (MTT method)

The concentration of H9C2 was adjusted to 1.0 × 10^4^ cells/mL after culturing, and then 200 μL of cell suspension was added to each well of a 96-well cell culture plate, and then adherent culture was carried out at 37°C for 24 h. After adherent cells are treated in two cases, first, 200 μL of MDLE medium solutions at concentrations of 0, 40, 100, 160 μg/mL were added to the wells. In the second case, before the MDLE treatment, the cells were first treated with 20 μL of hydrogen peroxide with a concentration of 0.3 mmol/L for 4 h, and then the cells were treated with the above concentration of MDLE medium solution. After 48 h, the cells were further treated with 200 μL of MTT reagent (Solarbio Life Science) at a concentration of 5 mg/mL for 4 h. Then, after all the medium was drained, 200 μL of sterile DMSO was added and shaken for 30 min. The final absorbance value was measured at 490 nm (Themo Genesys10s, ThermoFisher Scientific) (15).

#### MDLE affected the contents of MDA, SOD, GSH, GSH-Px, and CAT in H9C2 cells injured by oxidative stress

H9C2 cells in logarithmic growth phase were digested with 0.25% trypsin and adjusted to a concentration of (1 × 10^5^ cells/mL), then seeded into 6-well cell culture plates, and then 2 mL of DMEM medium was added to the wells, were incubated at 37°C for 24 h in 5% carbon dioxide. After adhesion, 200 μL hydrogen peroxide (0.3 mmol/L) was added, mixed, and cultured for 4 h to obtain the oxidative damage model. For the H9C2 oxidative damage cell model, 200 μL MDLE aqueous extract (0, 40, 100, and 160 μg/mL) was added to each well, and the final volume was adjusted with PBS buffer (0.1 M). Samples were incubated at 37°C, 5% CO_2_ in a saturated and humid environment for 24 h. After MDLE treatment, H9C2 cells were washed with pre-cooled PBS, dissociated with 200 μL trypsin, and transferred to a 1.5 mL centrifuge tube for supernatant removal. The cell pellet was washed again with pre-cooled PBS and centrifuged at 4,000 r/min for 15 min to remove the supernatant. The pellet was homogenized in 800 μL normal saline. The MDA (malondialdehyde), SOD (superoxide dismutase), CAT (catalase), GSH (glutathione), and GSH-Px (glutathione peroxidase) contents in cell homogenate were determined according to the instructions of the relevant kit (Solarbio Life Science) (16).

#### Flow cytometry

After MDLE treatment, 1 mL cell suspension of 5 × 10^5^ CFU/mL was centrifuged at 4,500 RPM and 4°C. The supernatant was discarded, and the cells were washed with pre-cooled PBS and suspended in 500 μL PBS. Cells were mixed with 5 μL annexin V-FITC and 5 μL propidium iodide (ThermoFisher Scientific) at 37°C in the dark for 15 min (AccuriC6, BD Biosciences, San Jose, CA, USA). Finally, flow cytometry was used for detection.

#### H_2_O_2_ effect on antioxidative genes expression in H9C2 cells

After the cells were cultured according to the previous experimental method, 1.0 mL RNAzol was added to extract the RNA from the tissue. Then measure the absorbance values of RNA extracts at 260 and 280 nm, and adjust the concentration of RNA to 1 μg/μL after calculating OD260/OD280. After reverse transcription, prepare a cDNA reaction system. The system solution includes cDNA (1 μL), SYBR Green PCR Master Mix (10 μL), upstream primers (1 μL, Table 1, ThermoFisher Scientific), downstream primers (1 μL) and sterile distilled water (7 μL). After the reaction solution was prepared, it was placed in a real-time quantitative PCR instrument, and under the set conditions (60 s at 95°C and 15 s at 95°C for 40 cycles, then 30 s at 55°C, 35 s at 72°C, 30 s at 95°C, 35 s reaction at 55°C) for mRNA amplification (SteponePlus, ThermoFisher Scientific), using GAPDH as the internal reference, and calculating the relative expression intensity of each gene according to the 2^−Δ*ΔCT*^ assay (17).

**Table 1:** The sequences of reverse transcription-polymerase chain reaction primers in this study.

**Gene name**	**Sequence**
*SOD*	Forward: 5′- AGATGGTGTGGCCGATGTGT-3′ Reverse: 5′- TCCAGCGTTTCCTGTCTTTGTA-3′
*CAT*	Forward: 5′- TGTTGCTGGAGAATCGGGTTC-3′ Reverse: 5′- TCCCAGTTACCATCTTCTGTGTA-3′
*GSH*	Forward: 5′- TACGGCTCACCCAATGCTC-3′ Reverse: 5′- CTATGGCACGCTGGTCAAATA-3′
*GSH-Px*	Forward: 5′- GTCGGTGTATGCCTTCTCGG-3′ Reverse: 5′- CTGCAGCTCGTTCATCTGGG-3′
*GAPDH*	Forward: 5′- TCA AGA AGG TGG TGA AGC AGG-3′ Reverse: 5′- AGC GTC AAA GGT GGA GGA GTG-3′

#### The main active components of MDLE were analyzed by high-performance liquid chromatography

MDLE extract was dissolved in DMSO to obtain a concentration of 10 mg/mL solution, then diluted with 50% methanol to obtain the final concentration of 2 mg/mL liquid phase sample through a 0.22 m organic filtration membrane test (injection volume: 10 μL). The Chromatographic column was an Accucore C18 column (2.6 μm, 4.6 × 150 mm), the Mobile phase A consisted of 0.5% acetic acid water, and the Mobile phase B consisted of acetonitrile. The flow rate was 0.6 mL/min, the column temperature was 35°C, and the detection wavelength was 254 nm. The Gradient elution conditions were 0–10 min in 12–25% B and 10–30 min in 25–45% B (UltiMate3000, ThermoFisher Scientific).

#### Statistical analysis of data

The experimental data were analyzed using SPSS 20.0 statistical software. Experimental results are expressed as mean ± standard deviation. Duncan's multi-range test was used to analyze the results by one-way analysis of variance (ANOVA), and *P* < 0.05 was considered to be statistically significant.

### Results

#### MDLE antioxidant test *in vitro*

**Figure 2:**
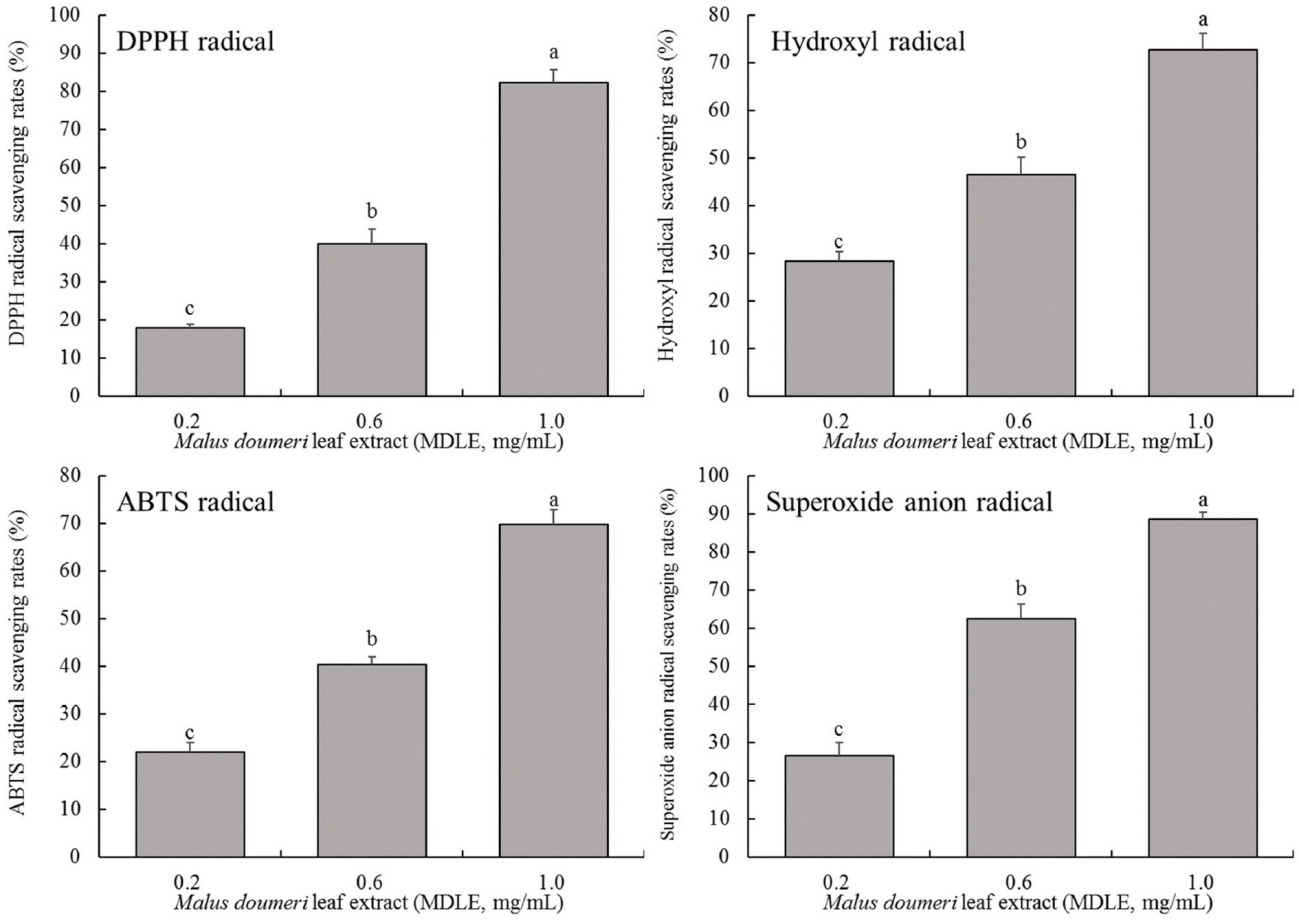
Hydroxyl radical, DPPH free radical, ABTS free radical and superoxide anion free radical scavenging rates of *Malus doumeri* leaf extract (MDLE). ^a−*c*^Lowercase letters are used to indicate significant differences at the *P* < 0.05 level. The same letter group indicates no difference, and the letter difference group indicates difference.

As shown in Figure 2, MDLE aqueous extract at concentrations of 0.2, 0.6, and 1.0 mg/mL significantly increased the scavenging rates of hydroxyl radical (28.4, 46.5, and 72.8%), DPPH free radical (31.9, 58.5, and 83.7%), ABTS free radical (22.0, 40.4, and 69.8%), and superoxide anion free radical (34.2, 62.7, and 87.6%). The results showed that MDLE had dose-dependent scavenging ability on four kinds of free radicals, and there were significant differences (*P* < 0.05).

#### H9C2 cell survival rate test (MTT method)

**Figure 3:**
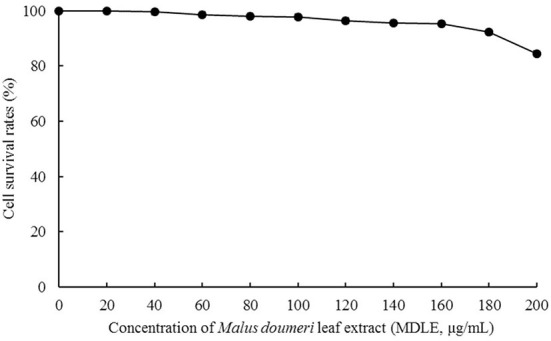
Effect of *Malus doumeri* leaf extract (MDLE) treatment on survival rate of H9C2 cells.

As shown in Figure 3, the survival rates of H9C2 cells were higher than 95% after MDLE treatments (0–160 μg/mL), indicating that MDLE within the concentration range (0–160 μg/mL) had no obvious lethal effect on H9C2. Therefore, 40, 100, and 160 μg/mL MDLE aqueous extracts were used for subsequent studies.

As shown in Table 2, H_2_O_2_ greatly reduced the survival rate of H9C2 cells after injury. After treatment with 80 and 160 μg/mL MDLE, the cell survival rate was significantly improved, and the protective effect of high concentration (160 μg/mL) was more pronounced (*P* < 0.05).

**Table 2:** Effect of *Malus doumeri* leaf extract (MDLE) treatment on survival rate of H_2_O_2_ induced oxidative damage of H9C2 cells.

**Group**	**Normal**	**Oxidative damage**	**H**_**2**_**O**_**2**_+**MDLE (**μ**g/mL)**	
			**80**	**160**
OD_490_	0.508 ± 0.005^a^	0.214 ± 0.009^d^	0.332 ± 0.008^c^	0.427 ± 0.007^b^
Survival rate (%)	100.00 ± 0.00^a^	42.09 ± 1.91^d^	65.30 ± 2.22^c^	84.13 ± 1.85^b^

a−d Lowercase letters are used to indicate significant differences at the P <0.05 level. The same letter group indicates no difference, and the letter difference group indicates difference.

#### MDLE treatment changes the MDA content in oxidative-damaged H9C2 cells

**Figure 4:**
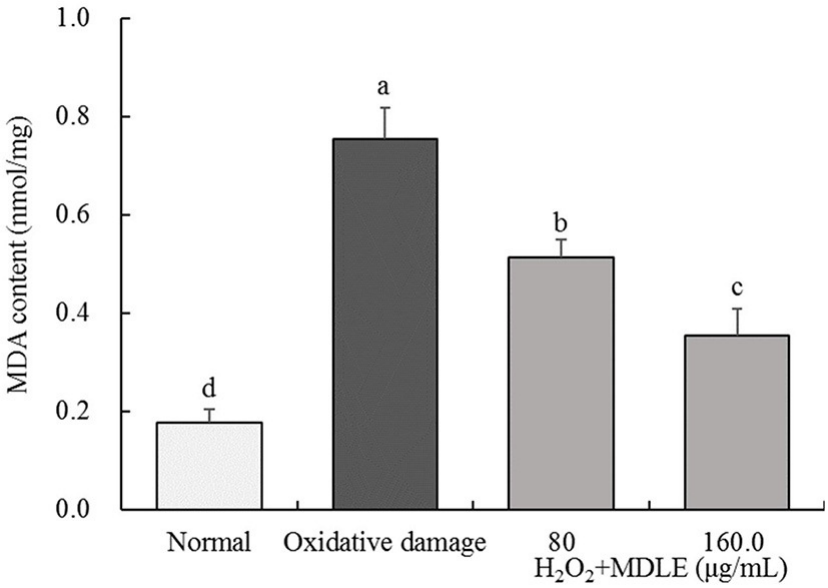
MDA content of *Malus doumeri* leaf extract (MDLE) treatment on H_2_O_2_ induced oxidative damage of H9C2 cells. ^a−*d*^Lowercase letters are used to indicate significant differences at the *P* < 0.05 level. The same letter group indicates no difference, and the letter difference group indicates difference.

As shown in Figure 4, after H_2_O_2_ treatment for 4 h, the MDA content of MDLE-treated H9C2 cells (0.76 ± 0.06 nmol/mg) was significantly higher than that of untreated cells (0.18 ± 0.03 nmol/mg). Different concentrations of MDLE aqueous extracts (80 and 160 μg/mL) significantly reduced the MDA content, and 160 μg/mL MDLE treatment had the lowest MDA content (0.36 ± 0.05 nmol/mg).

#### MDLE treatment changed the SOD, GSH, GSH-Px, and CAT contents in oxidative damaged H9C2 cells

As shown in Table 3, the SOD, GSH, GSH-Px, and CAT contents in H_2_O_2_-treated H9C2 cells (0.3 mmol/L) for 4 h were significantly lower than in normal cells. The cells' SOD, GSH, GSH-Px, and CAT contents were significantly improved after MDLE treatments (80 and 160 μg/mL), and the effect was the best in the 160 μg/mL MDLE treatment.

**Table 3:** SOD, GSH, GSH-Px, and CAT contents of *Malus doumeri* leaf extract (MDLE) treatment on H_2_O_2_ induced oxidative damage of H9C2 cells.

**Group**	**Normal**	**Oxidative damage**	**H**_**2**_**O**_**2**_+**MDLE (**μ**g/mL)**	
			**80**	**160**
SOD (U/mg)	7.82 ± 0.42^a^	3.23 ± 0.25^d^	5.02 ± 0.34^c^	6.16 ± 0.32^b^
GSH (μmol/mg)	0.79 ± 0.05^a^	0.33 ± 0.03^d^	0.48 ± 0.03^c^	0.63 ± 0.04^b^
GSH-Px (U/mg)	5.12 ± 0.26^a^	2.02 ± 0.21^d^	3.15 ± 0.26^c^	3.99 ± 0.16^b^
CAT (U/mg)	3.19 ± 0.23^a^	0.79 ± 0.18^d^	1.45 ± 0.20^c^	2.32 ± 0.21^b^

a−d Lowercase letters are used to indicate significant differences at the P <0.05 level. The same letter group indicates no difference, and the letter difference group indicates difference.

#### Effect of MDLE on apoptosis of oxidative-damaged H9C2 cells

**Figure 5:**
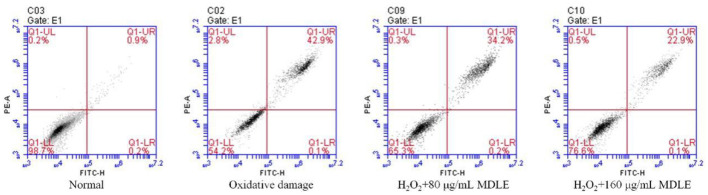
Apoptosis and death of H_2_O_2_ induced oxidative damage of H9C2 cells.

Apoptosis and death were induced in H_2_O_2_-treated H9C2 cells. MDLE significantly inhibit H_2_O_2_ induced oxidative H9c2 cell death, indicating that MDLE had an inhibitory effect on cell oxidative damage (Figure 5). And the inhibitory effect was positively correlated with the concentration.

#### MDLE affected *SOD, CAT, GSH*, and *GSH-Px* genes expression levels in damaged H9C2 cells

**Figure 6:**
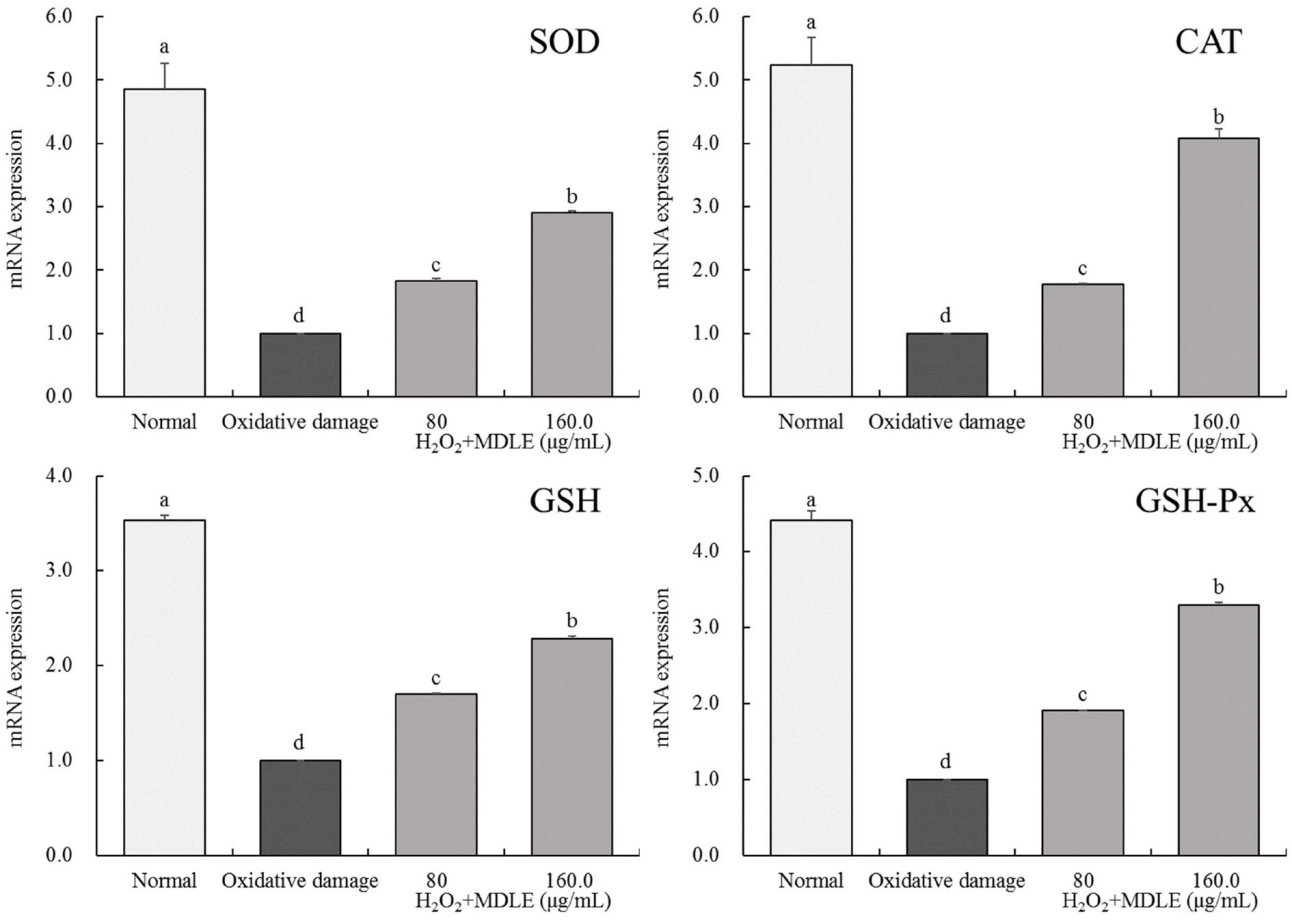
*SOD, CAT, GSH*, and *GSH-Px* mRNA expression of *Malus doumeri* leaf extract (MDLE) treatment on H_2_O_2_ induced oxidative damage of H9C2 cells. ^a−*d*^Lowercase letters are used to indicate significant differences at the *P* < 0.05 level. The same letter group indicates no difference, and the letter difference group indicates difference.

Real-time fluorescence quantitative PCR analysis shows that *SOD, CAT, GSH*, and *GSH-Px* mRNA expression levels in H9C2 cells were significantly decreased after H_2_O_2_ induced (0.3 mmol/L) injury, as shown in Figure 6. After MDLE treatment, the expression levels of endogenous antioxidant enzymes *SOD, CAT, GSH*, and *GSH-Px* in damaged cells were significantly increased (*P* < 0.05).

#### Liquid chromatography analysis of main components of MDLE

**Figure 7:**
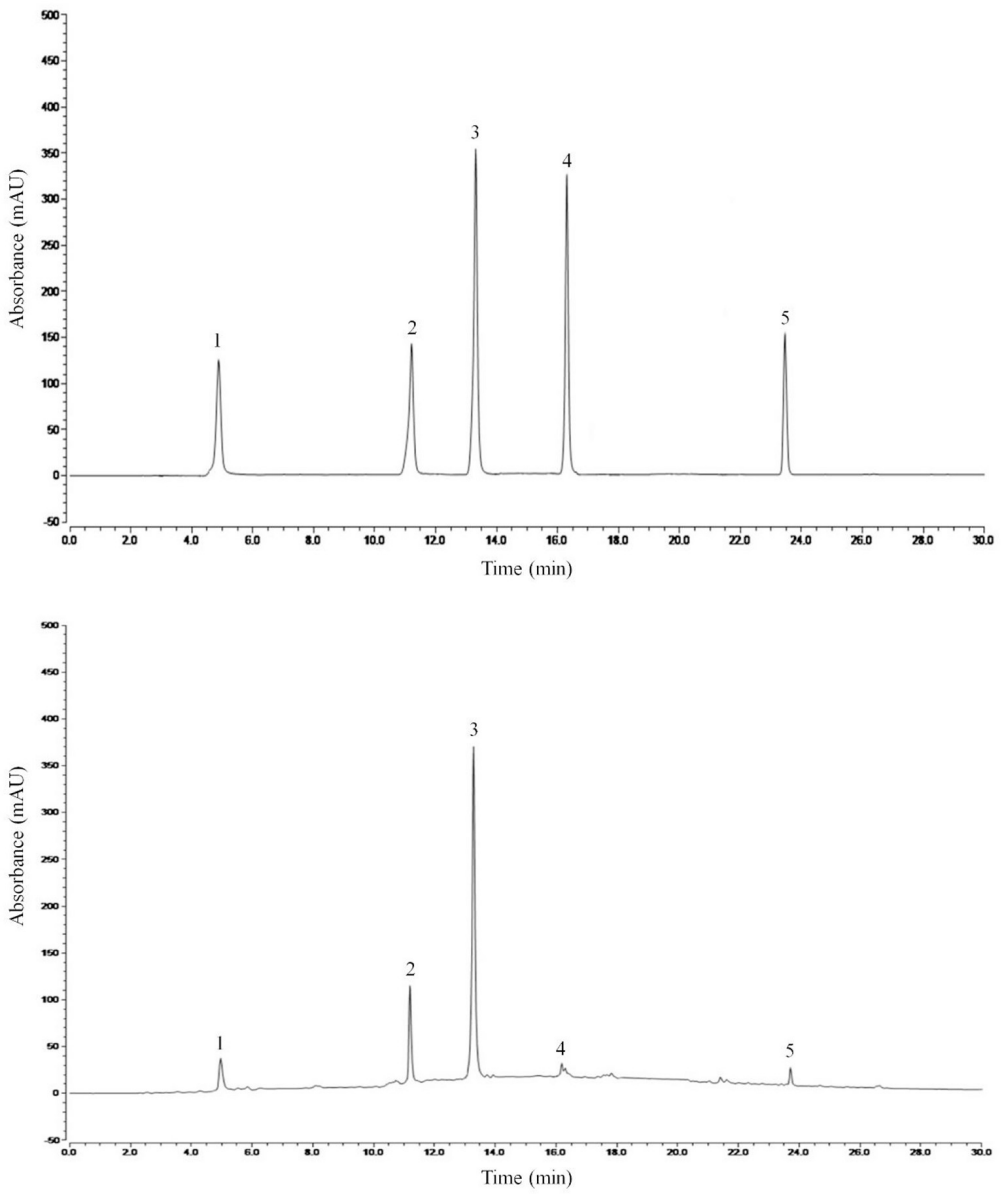
HPLC standard and *Malus doumeri* leaf extract chromatogram: 1, chlorogenic acid; 2, isoquercitrin; 3, quercetin; 4, baicalin; 5, phloretin.

Figure 7 shows the liquid chromatogram of MDLE and related standard substances. HPLC detected six compounds, including chlorogenic acid, isoquercitrin, quercetin, baicalin, and phloretin.

### Discussion

Oxygen-free radicals (OFR) can attack polyunsaturated fatty acids in biofilms and cause lipid peroxidation, which leads to oxidative damage to cells (18). There are two types of antioxidant systems in the body, which enable cells to maintain the redox homeostasis of free radicals and ROS. One is an enzymatic antioxidant system, including SOD, CAT, and GSH-Px; the other is a non-enzymatic antioxidant system, including antioxidants such as glutathione, vitamin C, vitamin E, coenzyme Q10, and alpha-lipoic acid. These antioxidants can neutralize free radicals and ROS, reduce the damage of free radicals and ROS to cells, and protect cells and tissues from oxidative damage (19). The body protects itself from damage caused by free radicals and ROS through a range of cell-intrinsic enzymatic and non-enzymatic antioxidants. These potentially harmful free radicals and ROS are regulated by the expression of endogenous antioxidants. The use of antioxidants helps to improve the body's antioxidant capacity, eliminate the harmful effects of free radicals and ROS, and prevent damage caused by a chain reaction of free radicals and ROS. *In vitro* studies confirm that various active components of plants can improve oxidative stress (20). DPPH is a stable free radical that becomes a stable yellow molecule after combining with electrons or hydrogen free radicals. It is one of the effective means to detect the antioxidant effect of substance extracts. The *in vitro* evaluation of the antioxidant activity of plant compounds or plant extracts is an important aspect of studying functional factors (21). Unbalanced levels of hydroxyl radicals in the body will cause oxidative damage to DNA, proteins and lipids and damage the body. The effect of antioxidants on oxidative damage can be detected by capturing •OH in a Fenton reaction system by salicylic acid (22). An oxidant can oxidize ABTS to produce blue-green ABTS•^+^; and the effect of antioxidants can make it fade (23). ${{\text{O}}_{2}}^{-}$ can be produced by the chain reaction of pyrogallol autooxidation system, and the color will be developed after o2- reaction ${{\text{O}}_{2}}^{-}$. The reaction attenuates its luminous signal (24). This study preliminarily confirmed that MDLE has antioxidant capacity in a dose-dependent manner through free radical scavenging experiments *in vitro*.

MDA is one of the important products of oxidative stress and lipid peroxidation. Its massive production and accumulation can cause serious damage to cell membranes, which in turn leads to cell death and damage to tissues and internal organs (25, 26). Hypoxia can cause myocardial tissue to generate a large number of oxygen free radicals (OFR). OFR acts on the unsaturated fatty acids on the cell membrane to produce peroxidation of membrane lipids, which in turn leads to myocardial cell damage and the formation of lipid peroxides. The important metabolites of OFR in the body can better reflect the degree of tissue peroxidation (27). After H9C2 cells were exposed to H_2_O_2_, the level of MDA increased, and MDLE could inhibit this occurrence, and the higher the dose, the better the effect. Superoxide dismutase is an active substance derived from living organisms, which can eliminate harmful substances produced by organisms in the process of metabolism, and has a special anti-aging effect on the human body by continuously supplementing SOD. SOD is one of the most effective antioxidants for scavenging free radicals. Its antioxidant capacity is 20 times that of vitamin C and 50 times that of vitamin E. Its specificity and efficiency in scavenging free radicals are unmatched by other antioxidants (28). Catalase is an antioxidant enzyme that is ubiquitous in almost all organisms, mainly in the liver and red blood cells of animals. It is the marker enzyme of peroxisome and accounts for about 40% of the total amount of peroxisomal enzymes (29). Glutathione peroxidase (GSH-Px) is an important peroxide-decomposing enzyme widely existing in the body, which can reduce toxic peroxides to non-toxic hydroxyl compounds, and at the same time promote the decomposition of H_2_O, thereby protecting the structure and function of the cell membrane from interference and damage by peroxides (30). Glutathione reductase can use NADPH to catalyze GSSG to produce GSH, and the activity level of glutathione peroxidase can be calculated by detecting the decrease of NADPH (31). MDLE can regulate the levels of CAT, SOD, GSH-Px and GSH in cells, thereby inhibiting lipid peroxidation and protecting cells.

Chlorogenic acid is a plant polyphenol that is often found in food. Studies have shown that it has a good antioxidant effect, which can also exert its anti-inflammatory effect (32). Isoquercetin is also a compound with antioxidant effects, and its physiological activities include anti-inflammatory, anti-viral, anti-tumor, and hypotensive (33). Quercetin is present in many plants, it is not only a good antioxidant, but also can lower blood sugar, and also has a certain effect on digestive system cancer (34). Baicalin is an active substance that can protect cardiovascular and cardiomyocytes, and can avoid heart damage (35). Phloretin is a kind of plant polyphenol with dihydrochalcone structure, which has antioxidant, anti-tumor, anti-inflammatory, immunosuppressive and other pharmacological effects (36). Some studies showed that *Malus doumeri* leaf from other places in China mainly contained 9 compounds, including the 5 compounds detected in this study (11, 37). In previous studies, the content of these 5 compounds was also high (38). Therefore, it could be considered that these 5 compounds were the main functional ingredients in *Malus doumeri* leaf from different places. Therefore, the presence of these bioactive chemicals can explain many pharmacological effects of MDLE.

## Conclusions

In conclusion, we evaluated the antioxidant activity of MDLE through *in vitro* experiments. We preliminarily analyzed the improvement effect and potential active compounds of MDLE under oxidative stress at the cellular level. MDLE had significant effects on DPPH, OH, ${{\text{O}}_{2}}^{-}$, and ABTS radical had significant scavenging activity. Meanwhile, the contents of CAT, SOD, GSH-Px and non-enzyme antioxidant GSH could be increased. Real-time quantitative PCR showed that MDLE could also up-regulate the transcription level of mRNA of related antioxidant enzymes to reflect the alleviating effect of oxidative stress. In addition, nine compounds, mainly flavonoids, were detected from MDLE by HPLC. These active compounds have been proved to have many biological activities. Finally, this study only carried out a preliminary study on the *in vitro* antioxidant activity and the effect of improving oxidative damage of *Malus doumeri* leaves, and the protective mechanism of its antioxidant effect needs to be further studied.

## Data availability statement

The original contributions presented in the study are included in the article/supplementary material, further inquiries can be directed to the corresponding author/s.

## Ethics statement

The animal study was reviewed and approved by the Affiliated Hospital of Guizhou Medical University.

## Author contributions

WL and DW conceived and designed the study. YS, ZS, PL, ZC, and BW conducted most of the experiments and data analysis and wrote the manuscript. DL, XS, and JP participated in collecting data and helped to draft the manuscript. All authors reviewed and approved the manuscript.

## Funding

This research was funded by Doctor Start-up Fund of Affiliated Hospital of Guizhou Medical University (gyfybsky-2021-54), the Health and Family Planning Commission of Guizhou Province (gzwkj2021-106 and gzwkj2021-273), the PhD Early Development Program of the Affiliated Hospital of Guizhou Medical University (gyfybsky-2021-33), Guizhou Science and Technology Support Plan: Qian Science and Technology Cooperation Support ([2020] 4Y231), Guiyang Science and Technology Plan ([2019]9-1-34), and Talent Team Project of Guizhou Provincial the Science and Technology innovation [qiankehepingtairencai(2020)5014: Talent team of Guizhou Province atherosclerotic disease prevention and treatment technology innovation].

## Conflict of interest

The authors declare that the research was conducted in the absence of any commercial or financial relationships that could be construed as a potential conflict of interest.

## Publisher's note

All claims expressed in this article are solely those of the authors and do not necessarily represent those of their affiliated organizations, or those of the publisher, the editors and the reviewers. Any product that may be evaluated in this article, or claim that may be made by its manufacturer, is not guaranteed or endorsed by the publisher.
